# Enhanced magneto-optical effects in composite coaxial nanowires embedded with Ag nanoparticles

**DOI:** 10.1038/srep29170

**Published:** 2016-07-11

**Authors:** Qianwen Liu, Xuanli Zheng, Jialun He, Weiping Wang, Mingming Fu, Yiyan Cao, Heng Li, Yaping Wu, Ting Chen, Chunmiao Zhang, Xiaohong Chen, Binbin Yu, Shuping Li, Junyong Kang, Zhiming Wu

**Affiliations:** 1 Fujian Provincial Key Laboratory of Semiconductors and Applications, Collaborative Innovation Center for Optoelectronic Semiconductors and Efficient Devices, Department of Physics, Xiamen University, Xiamen 361005, P. R. China; 2 Center for Nanophase Materials Sciences, Oak Ridge National Laboratory, Oak Ridge, Tennessee, 37831, USA

## Abstract

Nanostructures decorated with noble metal nanoparticles (NPs) exhibit potential for use in highly sensitive optoelectronic devices through the localized surface plasmon resonance (LSPR) effect. In this study, Faraday rotation was significantly enhanced through the structural optimization of ferromagnetic (FM)/semiconductor composite nanostructures. Experimental and theoretical results revealed that the position of noble metal NPs significantly influenced the coupling of the LSPR-enhanced electromagnetic field with FM materials. Furthermore, nanostructures embedded with noble metals demonstrated an improved capability to efficiently use the electromagnetic field compared to other structures. The Faraday rotation of ZnO/Ag(NPs)/Fe was enhanced 58 fold compared to that of the ZnO(film)/Fe. This work provides a basis for the design of nanoarchitectures for miniaturized high-performance magneto-optical devices.

The magneto-optical (MO) Faraday effect has attracted considerable research attention because of its potential applications in MO memory systems, biosensors, and optical isolators^1^,^2^,^3^,^4^. Physically, the Faraday effect arises from the interactions between optical radiation and matter in the presence of a magnetic field; thus, the intensity and distribution of the light field inside the material are major factors that affect device performance^5^,^6^,^7^. However, the MO Faraday effect in common ferromagnetic (FM) films is insufficiently strong for device applications^8^. Therefore, researchers have focused on improving the FM performance through structural optimization^5^,^9^,^10^,^11^,^12^,^13^. Nanowire (NW) arrays are preferred and widely used for this optimization because of their low reflectance and cavity effect^14^,^15^,^16^. Additionally, nanostructures decorated with noble metals have been extensively introduced to enhance the Faraday effect through surface plasmon polariton (SPP) or localized surface plasmon resonance (LSPR) effects^5^,^11^,^12^,^17^,^18^,^19^,^20^,^21^,^22^,^23^,^24^,^25^. These effects generate strong light scattering and intensive absorption bands and also enhance the local electromagnetic fields^26^. For example, Belotelov *et al*.^24^ fabricated an MO material consisting of a nanostructured gold film on top of an FM dielectric and reported SPP-enhanced MO effects. Jain *et al*.^23^ reported the LSPR-enhanced Faraday rotation in gold-coated maghemite nanoparticles (NPs). Hence, it is quite reasonable to believe that a combination of NW structures and noble metal decoration could enhance the MO effect. However, SPP and LSPR effects of noble metals are easily affected by the size, shape, and the medium that surrounds or is near the metallic structure, and the additional deposition of metals may cause the damping of plasmon oscillations^27^. To minimize the influence of adjacent media in common composite structures, most studies have adopted structures with noble metal particles or thin films on the outer layer of the FM material^23^,^27^,^28^. In these cases, the SPP or LSPR effect is relatively strong, but the enhanced surface electromagnetic field cannot effectively interact with the FM materials. Therefore, increasing the utilization ratio of the surface electromagnetic field induced by the SPP or LSPR effect remains a challenge.

In this work, ZnO/Fe, ZnO/Fe/Ag(NPs), and ZnO/Ag(NPs)/Fe coaxial NWs were fabricated through chemical vapor deposition (CVD) and magnetron sputtering. The effects of the structures on the enhancement of the Faraday effect were investigated. The results show that the strengthened electromagnetic field mainly exists in the outer surface of Ag NPs when the noble metals are located outside the FM materials, resulting in a low field coupling between the Ag NPs and the Fe layer. Nevertheless, the embedded nanostructures of noble metal NPs increase the effective utilization of the LSPR-enhanced electromagnetic field, thereby exhibiting the maximal MO effect. A new enhancement mechanism was proposed and assessed through experimentation and theoretical simulation.

## Results

**Figure 1:**
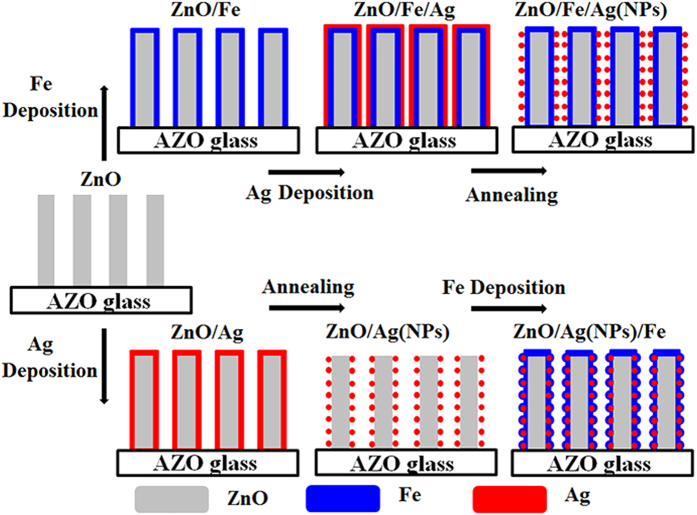
Schematic of the formation of coaxial nanostructures.

**Figure 2:**
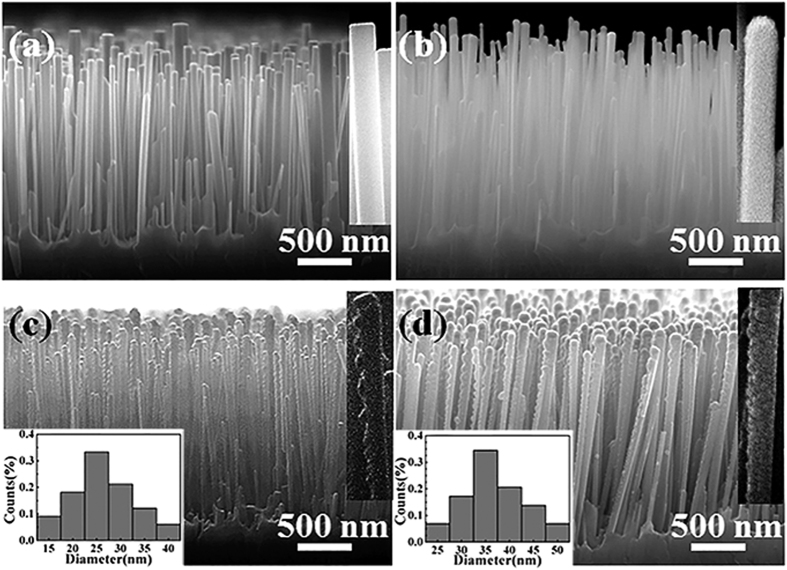
Cross-sectional SEM images. (**a**) Bare ZnO NWs, (**b**) ZnO/Fe NWs, (**c**) ZnO/Fe/Ag(NPs) NWs, and (**d**) ZnO/Ag(NPs)/Fe NWs. Insets at top right corner in (**a–d**) display the typical high-magnification SEM images of a single NW. Insets at lower left corner in (**c,d**) show histograms of particle diameter distributions in the ZnO/Fe/Ag(NPs) and ZnO/Ag(NPs)/Fe NWs.

In this experiment, three types of coaxial NWs, i.e., ZnO/Fe, ZnO/Fe/Ag(NPs), and ZnO/Ag(NPs)/Fe, were fabricated. The schematic of the fabrication process is presented in Fig. 1. Figure 2(a) shows the scanning electron microscopy (SEM) image of the cross-sectional view of ZnO NWs, featuring vertical growth and a smooth surface (inset). After the deposition of different shells [Fig. 2(b–d)], the NW surfaces coarsen, indicating the formation of a coaxial structure. Notably, ZnO/Fe/Ag(NPs) and ZnO/Ag(NPs)/Fe NWs possess granular surfaces [Fig. 2(c,d), respectively]. Statistical analyses of particle size for ZnO/Fe/Ag(NPs) and ZnO/Ag(NPs)/Fe NWs were performed. Insets at lower left corners in Fig. 2(c,d) show the histograms of particles size distribution. The size of the Ag particles in the ZnO/Fe/Ag(NPs) nanowires shows a Gaussian distribution with a peak at 25 nm. More than 70% particles have a diameter in the 20–35 nm range, demonstrating relatively uniform particles. As shown in Fig. 2(d), for the ZnO/Ag(NPs)/Fe NWs, the average particle size increased owing to the Fe layer deposition. The formation of NPs is mainly attributed to the shrinking of the Ag film during thermal annealing^29^. Insets at top right corners in (a–d) display the typical high-magnification SEM images of a single NW. The entire nanowire was almost coated by the outside layer (seen in inserts of Fig. 2(c,d) at the top right corners). We predicted that the special structure could provide novel MO properties.

**Figure 3:**
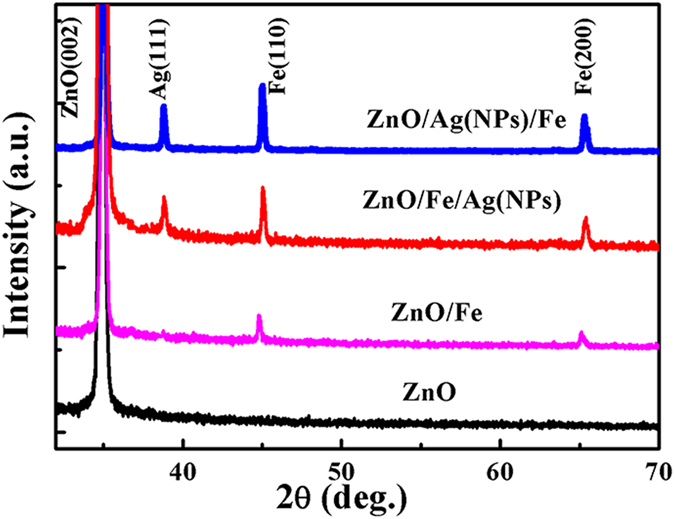
XRD patterns of different samples.

To investigate the structures of different samples, X-ray diffraction (XRD) analysis was performed. As shown in Fig. 3, for bare ZnO NWs, a sharp peak appears at 34.8°; this peak can be indexed to the (002) plane of wurtzite (WZ) ZnO. After the deposition of the Fe shell layer, two additional characteristic peaks appear at 44.6° and 64.9° in the ZnO/Fe NWs, corresponding to the (110) and (200) planes of Fe, respectively. Furthermore, a distinct peak at 38.2°, assigned to the (111) Ag planes, is observed for the ZnO/Fe/Ag(NPs) and ZnO/Ag(NPs)/Fe NWs. These characteristic peaks match well with the nominal compositions of the coaxial NWs, thereby indicating the successful fabrication of multi-shell coaxial NWs.

**Figure 4:**
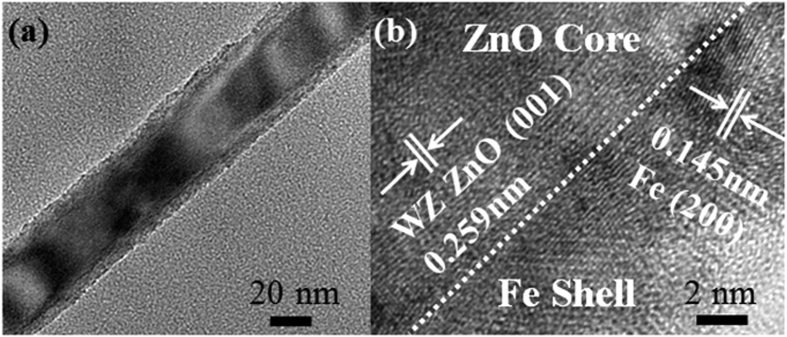
TEM images. (**a**) Low-resolution TEM image and (**b**) HRTEM image of ZnO/Fe NW.

**Figure 5:**
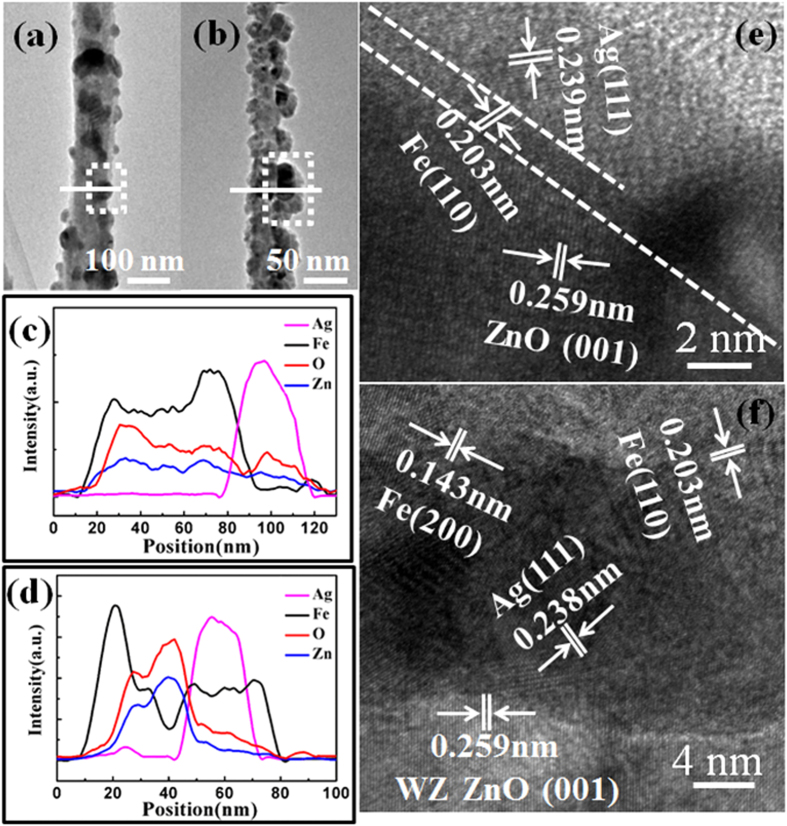
TEM images. (**a**) ZnO/Fe/Ag(NPs) NW and (**b**) ZnO/Ag(NPs)/Fe NW. (**c,d**) EDS line-scan profiles along the lines marked in (**a,b**), respectively. HRTEM images of (**e**) ZnO/Fe/Ag(NPs) NW and (**f**) ZnO/Ag(NPs)/Fe NW taken from the area marked by white squares in Fig. 5(a,b), respectively.

To characterize the interior structures of the ZnO/Fe, ZnO/Fe/Ag(NPs), and ZnO/Ag(NPs)/Fe NWs, transmission electron microscopy (TEM) and energy-dispersive X-ray spectroscopy (EDS) analyses were conducted. Figure 4(a) shows the low-magnification TEM image of the ZnO/Fe NW. A uniform shell layer covers the ZnO core, demonstrating the successful fabrication of the shell layer through magnetron sputtering. Figure 4(b) displays the high-resolution TEM (HRTEM) image of the interfacial region in the ZnO/Fe NW. The upper left section indicates WZ ZnO with the [001] growth direction, whereas the lower right section corresponds to Fe with an interplanar spacing of 0.145 nm and [200] growth direction. These findings confirm the successful fabrication of the ZnO/Fe coaxial NW. Figure 5(a,b) show the TEM images of the ZnO/Fe/Ag(NPs) and ZnO/Ag(NPs)/Fe NWs, respectively, that possess granular surfaces, in contrast to the ZnO/Fe NW in Fig. 4(a). For the ZnO/Fe/Ag(NPs) NW, NPs are located on the outermost surface, and the morphology of this structure assumes a semi-spherical shape. The EDS line-scan profile shown in Fig. 5(c) reveals the Ag composition of the NPs. Notably, the size of the Ag NPs follows a certain distribution pattern because of the random shrinkage during annealing. In the experiment, the annealing temperature was set at 450 °C to maintain an average particle size of approximately 25 nm, as discussed in Fig. 2. For the ZnO/Ag(NPs)/Fe NW [Fig. 5(b)], the size of the NPs increases and the surface coarsens because of the changes in the coverage of the Fe shell compared to that in Fig. 5(a). Figure 5(d) shows the EDS line-scan profile along the line marked in Fig. 5(b). Ag is located inside the NW. To elucidate the structures of NPs, HRTEM analysis of the cross-sectional images was conducted. As shown in Fig. 5(e), a uniform Fe layer is deposited on the ZnO core, and semi-spherical Ag NPs are located outside the Fe layer in the ZnO/Fe/Ag(NPs) NW. For the ZnO/Ag(NPs)/Fe NW [Fig. 5(f)], the Fe layer completely covers the Ag NPs, and a coaxial structure with embedded Ag NPs is formed. The differences between the structures of these two NWs significantly influence their magnetic and optical properties.

**Figure 6:**
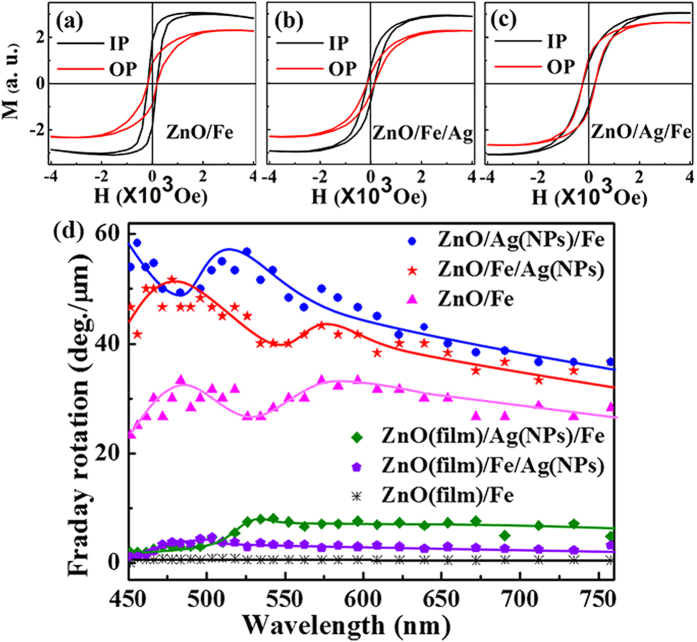
Magnetic properties of samples. Room-temperature hysteresis loops of (**a**) ZnO/Fe NWs, (**b**) ZnO/Fe/Ag(NPs) NWs, and (**c**) ZnO/Ag(NPs)/Fe NWs; and (**d**) Faraday effect of different samples. Incident light was perpendicular to the sample surface.

Figure 6(a–c) show the room-temperature in-plane (IP, with the magnetic field parallel to the substrate surface) and out-of-plane (OP, with the magnetic field perpendicular to the substrate surface) hysteresis loops for different coaxial NWs. The measured coercivity (H_*c*_) and squareness (saturation remanence divided by saturation magnetization, M_r_/M_s_) values are summarized in Table 1. The H_*c*_ values of all samples are relatively small, indicating the good soft magnetic property of the Fe layer. H_*c*_ slightly increases in the ZnO/Ag(NPs)/Fe NWs compared with that in the ZnO/Fe NWs, owing to the pinning effect of Ag NPs^30^. However, H_*c*_ decreases in the ZnO/Fe/Ag(NPs) NWs. This anomalous phenomenon may be attributed to the counteracting effect of mismatch stress in sandwich-like structures^31^,^32^. Furthermore, M_r_/M_s_ shows a varied degree of changes when Ag NPs are embedded on NWs. For the ZnO/Fe NWs, the high M_r_/M_s_ of the IP loops (square shape) indicates that the easy axis of magnetization is along the IP direction. Generally, in this case of ZnO/Fe NWs with a thin Fe layer, a relatively strong demagnetizing field is formed along the in-plane direction, favoring the longitude magnetic structure (OP direction). However, the strong orbital hybridization of Fe and O elements at the interface is beneficial for the perpendicular magnetic anisotropy (i. e., IP direction)^33^. Hence, it is believed that the latter plays the major role in the formation of the magnetic structure in ZnO/Fe NWs. Additionally, for ZnO/Fe/Ag(NPs) and ZnO/Ag(NPs)/Fe NWs, M_r_/M_s_ decreases in various degrees; therefore, the easy axis of magnetization tends to rotate to the OP direction because of the effect of Ag NPs.

Figure 6(d) shows the Faraday effect in different samples. The Faraday effects in the samples based on ZnO films are relatively small (less than 10 degree/μm) and are almost undistinguishable from those in ZnO(film)/Fe. However, upon the adoption of the NW structure, the Faraday effect was obviously enhanced in various degrees. The Faraday rotation angle in ZnO/Fe NWs is approximately 33 degree/μm at the wavelength of 480 nm, almost 33 times greater than that of ZnO(film)/Fe. This enhancement may be attributed to two factors. On the one hand, the NW structure has a longer optical path in the longitudinal direction compared to the film structure, resulting in the increased Faraday rotation angle. On the other hand, a cavity is formed in the ZnO NWs, and the multiple reflections of the light in the cavity enhance the interaction between the light and the Fe layer^8^, accordingly enhancing the Faraday effect. As shown in Fig. 6(d), an oscillating behavior appears in the Faraday curves of the NW samples. Considering the fact that the light reflection in ZnO/Fe and ZnO/Fe/Ag(NPs) NWs occurs at the ZnO-Fe layer interfaces, it is not surprising that their curves show similar shapes owing to the similar cavity parameters. Nevertheless, for the ZnO/Ag(NPs)/Fe NWs, the oscillating peak exhibits a certain blue-shift^27^. Furthermore, it is worth noting that the NW samples decorated by Ag NPs, i.e., ZnO/Fe/Ag(NPs) NWs and ZnO /Ag(NPs) /Fe NWs, show larger Faraday effects than the ZnO/Fe NW. The maximal rotation angles in the ZnO/Fe/Ag(NPs) NWs and ZnO/Ag(NPs)/Fe NWs are, respectively almost 52 and 58 times greater than that in the ZnO(film)/Fe. This is believed to originate from the LSPR effect induced by the Ag NPs^29^. The enhanced electromagnetic field due to the LSPR effect favors the interaction of the light and the FM material, and thus improves the Faraday effect. Here, it should be mentioned that no obvious enhanced peaks can be assigned to the LSPR effect of Ag NPs, which may be attributed to the Fe capping layer. The outer Fe layer absorbs or reflects some of the light, and then makes the light that reaches Ag NPs decrease to a certain extent. Meanwhile, the Fe capping layer may cause the damping of plasmon oscillations^27^. As a result, the LSPR effect weakens and the resonance peak becomes indistinct. Interestingly, the ZnO/Ag(NPs)/Fe NWs exhibit a stronger Faraday effect than the ZnO/Fe/Ag(NPs) NWs. In this sense, the position of Ag NPs in the composite materials also affects the Faraday effect enhancement. As we know, the Faraday effect is concerned with the interaction between light and matter and strongly depends on the distribution of the light field inside a material. To explore the intrinsic mechanism, the absorption spectra were measured and the electromagnetic field distribution was simulated as well.

**Figure 7:**
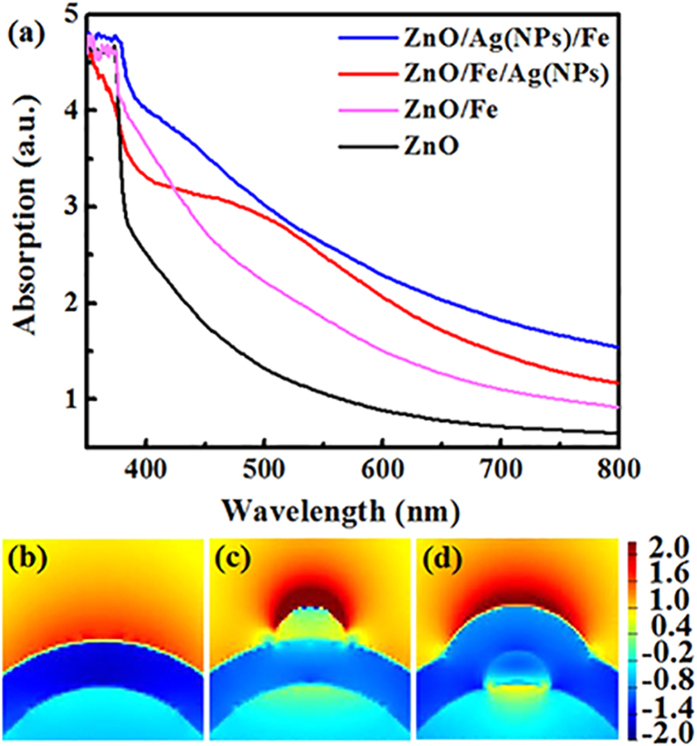
UV-Vis absorption spectra and FDTD simulating results. (**a**) UV-Vis absorption spectra of pure ZnO, ZnO/Fe, ZnO/Fe/Ag(NPs), and ZnO/Ag(NPs)/Fe NWs. Simulated near-field distributions of (**b**) ZnO/Fe NWs, (**c**) ZnO/Fe/Ag(NPs), and (**d**) ZnO/Ag(NPs)/Fe NWs.

Figure 7(a) displays the UV-Vis absorption spectra of different samples. ZnO NWs reveal a sharp band edge absorption at approximately 380 nm. After the deposition of the Fe shell (i.e., ZnO/Fe NWs), the absorption edge does not shift; however, the absorption over the entire visible range increases because of the light loss inside the Fe materials. Interestingly, the subsequent decoration of Ag NPs further enhances light absorption. Moreover, the enhancement behavior varies with the position of Ag NPs. For ZnO/Fe/Ag(NPs), a broad absorption peak occurs at ~500 nm, that could be attributed to the Ag-induced LSPR effect^26^. The LSPR peak position depends significantly on the size of metal NPs, that is, a sharp peak can be observed for uniform-sized NPs. In the experiment, Ag NPs exhibit a wide size distribution, resulting in the broadening of the absorption peak (Fig. 2). By contrast, when Ag NPs are embedded in coaxial NWs, i. e., ZnO/Ag(NPs)/Fe, the absorption peak disappears, possibly owing to the additional Fe capping layer weakening the LSPR, as discussed in Fig. 6(d). Nevertheless, the improved light absorption performance is observed over the entire visible range. To elucidate the mechanism, near-field distributions of ZnO/Fe, ZnO/Fe/Ag(NPs) and ZnO/Ag(NPs)/Fe NWs at *λ* = 500 nm were simulated using the finite-difference time domain (FDTD) method. As shown in Fig. 7(b,c), the local field of coaxial NWs with Ag NP decoration is stronger than that of ZnO/Fe NWs because of the LSPR effect, contributing to the increased light absorption. Notably, the enhanced electric field in the ZnO/Fe/Ag(NPs) NWs is mainly concentrated on the top of Ag NPs, and the field coupling between Ag and Fe is relatively weak. For ZnO/Ag(NPs)/Fe NWs, the field intensity near Ag NPs is obviously weakened because of the Fe capping layer, resulting in the disappearance of the resonance absorption peak shown in Fig. 7(a). However, the intensity of the field surrounding the Fe layer, especially on its surface, drastically increases, thereby contributing to the enhancement of light absorption. This behavior reveals that Ag-NP-embedded nanostructures exert a stronger coupling effect between the Fe layer and Ag NPs than the other structures. This attribute is more beneficial for strengthening the interactions between the light field and the Fe layer, eventually contributing to improvement of the MO effect.

## Discussion

The influence of material structure on the LSPR-enhanced MO Faraday effect in composite coaxial NWs was investigated, with a particular focus on the coupling of a LSPR-enhanced electromagnetic field with FM materials. Morphological and structural studies using SEM, XRD, and TEM analyses demonstrated the successful fabrication of three types of coaxial NWs, i.e., ZnO/Fe, ZnO/Fe/Ag(NPs), and ZnO/Ag(NPs)/Fe NWs. Measurement of hysteresis loops revealed that while the magnetic structure of the FM layer changed slightly with the position of Ag NPs, the suitable soft magnetic properties were retained. Importantly, MO studies and FDTD simulations showed that the introduction of Ag NPs considerably enhanced the Faraday effect owing to the LSPR effect. Moreover, the position of the Ag NPs significantly influenced the coupling of the LSPR-enhanced electromagnetic field with the FM material. ZnO/Ag(NPs)/Fe NWs embedded with Ag NPs showed an improved capability to efficiently use the electromagnetic field compared to the other NWs. As a result, the Faraday rotation was enhanced 58 fold compared to that of the ZnO/Fe(film). This work provides guidance in the design of novel nanoarchitectures for miniaturized high-performance MO devices.

## Methods

In the experiment, ZnO NWs used as a template were grown using the CVD method, described in detail in our previous work^34^. Fe shells with a thickness of ~10 nm were deposited by radio frequency magnetron sputtering of the Fe (99.99%) target in ambient Ar. The distance between the target and the substrate was set to 8 cm. Prior to the deposition, the vacuum chamber was evacuated down to 4.0 × 10^−4^ Pa. During the growth, the working pressure was maintained at 1.0 Pa, and the sputtering power was set at 150 W. Ag thin films with a 15 nm thickness were fabricated through direct current magnetron sputtering of the Ag (99.99%) target. The growth pressure was maintained at 0.8 Pa, and the sputtering power was set as 60 W. Thermal treatment was then conducted through rapid thermal annealing at 400 °C for 2 min to fabricate Ag NPs. Additionally, a ZnO (film)/Fe sample was fabricated for comparison; a ZnO film with a thickness of 100 nm was grown using molecular beam epitaxy at room temperature.

The morphologies of the as-prepared ZnO, ZnO/Fe/Ag(NPs), and ZnO/Ag(NPs)/Fe NWs were characterized by field-emission SEM (LEO 1530, operated at 20 kV). The structures and composition of these NWs were analyzed using XRD (Rigaku Ultima IV), TEM (Tecnai F30, operated at 300 kV), and EDS. The absorption spectra were obtained using a Varian Cary 5000 UV-Vis NIR spectrophotometer. The magnetic properties were studied using a vibration sample magnetometer (Quantum Design). The MO Faraday rotation was measured using a home-made system. The electric fields of coaxial NWs were simulated using the three-dimensional FDTD method.

## Additional Information

**How to cite this article**: Liu, Q. *et al*. Enhanced magneto-optical effects in composite coaxial nanowires embedded with Ag nanoparticles. *Sci. Rep*. **6**, 29170; doi: 10.1038/srep29170 (2016).

## Figures and Tables

**Table 1:** In-plane and out-of-plane magnetic properties.

	IP		OP	
	H_*c*_(Oe)	M_r_/M_s_	H_*c*_(Oe)	M_r_/M_s_
ZnO/Fe	155	0.603	180	0.386
ZnO/Fe/Ag(NPs)	124	0.243	100	0.175
ZnO/Ag(NPs)/Fe	240	0.371	251	0.343
